# DAILY WORK-TO-FAMILY CONFLICT AND SELF-REPORTED COGNITIVE HEALTH IN MIDLIFE

**DOI:** 10.1093/geroni/igae098.1977

**Published:** 2024-12-31

**Authors:** Kelly Chandler, Madeline Nichols, Robert Stawski

**Affiliations:** Oregon State University, Corvallis, Oregon, United States; Oregon State University, Corvallis, Oregon, United States; University of Essex, Colchester, England, United Kingdom

## Abstract

Work-family conflict (WFC) is a potent stressor in midlife linked to numerous detrimental physical and mental health outcomes; however, less is known about its effects on cognitive health. Increasing research demonstrates the utility of intensive repeated measures designs to examine within-person variability in WFC and health. Therefore, the aims of the present study were (a) to examine within-person associations between daily WFC and cognitive interference and memory failures and (b) to test age and gender as moderators. The data come from 131 IT employees who participated in an eight-day daily diary in the Work, Family, & Health Study (Age M=45, SD=6.33; 45% women). We found both significant between- and within-person associations from our two-level multilevel models. On average, adults who had higher WFC experienced more cognitive interference, Est.=0.22, SE=.06, p<.001, and memory failures, Est.=0.41, SE=.09, p<.001. On days when adults experienced higher WFC than was typical for them, they experienced more cognitive interference, Est.=.10, SE=.03, p<.01, and memory failures, Est.=0.18, SE=.06, p<.01. Age moderated the association between average WFC and memory failures. Simple slopes tests showed significant positive associations for adults aged 35, Est.=0.86, SE=.20, p<.001, and 45 (the mean), Est.=2.15, SE=.70, p<.01, but not 55, Est.=0.11, SE=.15, p=.43. Gender was not a significant moderator. These preliminary results indicate that there is day-to-day variability in WFC and self-reported cognitive health, particularly for adults in their 30s and 40s. Learning more about days with high WFC could suggest strategies for daily interventions to minimize the impacts of WFC on cognitive health.

